# Maximizing Coverage Quality with Budget Constrained in Mobile Crowd-Sensing Network for Environmental Monitoring Applications

**DOI:** 10.3390/s19102399

**Published:** 2019-05-26

**Authors:** Jiaoyan Chen, Jingsen Yang

**Affiliations:** The School of Information Engineering, Nanchang University, Nanchang 330047, China

**Keywords:** coverage, mobile crowd-sensing network, environmental monitoring, limited budget

## Abstract

The Mobile Crowd-sensing Network is a novel cyber–physical–social network which has received great attention recently and can be used as a powerful tool to monitor the phenomenon of the field of interest. Due to the limited budget, how to choose appropriate participants to maximize the coverage quality is one of the most important issues when the mobile crowd-sensing network applies to practical application, such as air quality monitoring. In this paper, given the number of available participants, the traverse path and the reward of each participant, we investigate the problem of how to choose suitable participants to monitor an environment of a critical region by a crowd-sensing network, while the total rewards for all selected participants is not larger than the limited budget. In our solution, we first divide a big critical region such as a city into smaller regions of different size, and select some sampling points in the smaller region; the collected data of those sampling points represents the collected data of the whole smaller region. Then, we design a greedy algorithm to select participants to cover the maximum sampling points while the total rewards of selected participants does not exceed the limited budget. Finally, we evaluate the validity and efficiency of the proposed algorithm by conducting extensive simulations. The simulation results show that the greedy algorithm outperforms an existing scheme.

## 1. Introduction

A Cyber–Physical System (CPS) is an intelligent system that integrates computing, network and physical environments, it can realize real-time perception and dynamic control of large systems through deep cooperation of 3C technology (Computation, Communication and Control) [1,2]. With the development of computing systems, a Cyber–Physical–Social System (CPSS) allows humans to participate in the system not only as service consumers but also as information providers. The Mobile Crowd-Sensing Network (MCSN) is a novel cyber–physical–social system. In MCSN, humans carry smart devices with sensing and wireless communication capabilities which allow them to collect and upload data to the assessment center for large-scale sensing and community intelligence mining [3,4,5,6,7,8,9,10].

To be specific, a mobile crowd-sensing network consists of mobile smart devices like mobile phones and assessment center. The mobile smart devices carried by mobile population are embedded with some low cost, low power and reliable micro sensors [11], which can sense the physical information at some specific locations and ensure that the data collected by sensors are not distorted [12,13]. After assessment center initiates the sensing tasks, it will analyze the data uploaded by the mobile population [14,15]. Mobile crowd-sensing network has been widely used in real life as a powerful tool to monitor the phenomenon of the field of interest, such as map semantic labeling [16,17,18,19,20], road condition monitoring [21,22,23], noise monitoring [24,25,26], crowdsourcing images [27,28] and pollution monitoring [29,30].

Data acquisition from a critical region for environmental monitoring applications is an important application of mobile crowd-sensing network. For instance, collecting air data or noise data to monitor the air quality or noise level of the region. Manu people can schedule their activities according to the environmental quality of some particular regions since it is harmful to take part in activities in the region with poor environmental quality. There are lots of studies about how to complete the data acquisition task, and most of those works use traditional wireless sensor network (WSN) [31,32,33,34,35,36,37,38,39] or vehicle sensor network (VSN) [40,41,42,43]. Wireless sensor network collects the information and transmits the data to the server through wireless transmission by deploying some wireless sensors in critical region. It provides continuous monitoring by collecting the data constantly. Vehicle sensor network places sensors inside the car to collect information at different locations while the car is moving. It also provides stable and long-term monitoring when the vehicles carried sensor move periodically inside the region.

Compared to the crowd-sensing network, the wireless sensor network has the following disadvantages. First, the wireless sensors are battery-powered which have fixed sensing radius and finite lifetime. The network designer should replace the battery frequently, while the smart devices in a crowd-sensing network can be powered by portable power source and the sensing radius varies with the sensing power. Secondly, the deployment locations of wireless sensors are fixed and whole wireless sensor networks which can not move to different critical regions. On the contrary, the crowd-sensing network is more flexible and the assessment center only needs to select some suitable data collectors that are active inside the critical region. Thirdly, the total deployment cost of wireless sensor networks is larger than a crowd-sensing network since the former one needs to buy sensors to construct network and repair them if they are out of work, let alone the expense of labour to maintain the network [44,45,46]. While the crowd-sensing network selects different qualified participants under the condition that the total rewards of participants is no larger than the given budget.

With the above-mentioned advantages, we investigate the problem of how to monitor the information of a critical region by mobile crowd-sensing network in this paper, in order to accurately obtain a large number of dynamic data in space, we propose a data acquisition method in the mobile crowd-sensing network. The solution to data acquisition in the crowd-sensing network should consider two aspects. First, the assessment center should take sampling in some places inside the region and the number of such sampling places can be large. However, the air quality of two space points not far from each other in a critical region does not change much, so we choose some locations inside the region as sampling points and the collected data of those sampling points can represent the collected data of the whole critical region. Therefore, the first aspect to be considered is how to choose the sampling points. The second aspect is the reward of the participants. Once the sampling points are determined, the assessment center should select participants to cover all sampling points in order to collect the data. However, the participants do not collect and send data for free since it takes extra cost and time for participants to sense and send the data to the assessment center. Therefore, the assessment center should give certain reward to participants. Furthermore, the assessment center has a limited budget for each critical region. In our work, we assume the reward of each participant is put forward by themselves. The key of the second aspect is how to select the suitable participants to achieve the maximum coverage quality under the limit of budget.

In our work, we first present a network model and describe the coverage model which is suitable to monitor the interest of a field of the whole region. First, we divide a big critical region into multiple small rectangles with the same size. The center of the rectangle is selected as the sampling point and the collected data of such a point represents the collected data of the small rectangle. Note here, the number of the rectangles depends on the monitoring requirement and geographical characteristics of the critical region. The higher monitoring requirement and rugged region need more rectangles and in turn more sampling points in the network. Let *m* denote the number of the sampling points in the critical region. Those sampling points have the same contribution as the environmental monitoring. In order to monitor the critical region environment, the selected participants should collect data from all sampling points. Therefore, the goal of the paper is to select participants whose traverse paths can cover maximum sampling points in the critical region. Once we determine the locations of *m* sampling points, we solve the second problem that chooses suitable participants to cover the sampling points. Given the number of candidate participants, their traverse paths, the reward of each participant and the budget afforded by the assessment center, a greedy algorithm is designed to select the participants with the objective of maximizing the coverage quality while the total rewards of selected participants is no larger than the budget.

The rest of the paper is organized as follows. Section 2 reviews related work. The network model, communication model and problem description are presented in Section 3. In Section 4, we design greedy algorithm to solve the MCLB problem. We evaluate the proposed algorithm by simulations and analyze the simulation results in Section 5. Finally, we conclude our work in Section 6.

## 2. Related Work

Many coverage problems in mobile crowd-sensing networks have been studied in recent years, and two main problems have been considered: how to improve the coverage quality [47,48,49,50] and save energy while maintain the coverage quality [51,52,53,54].

The coverage quality is a critical issue in a mobile crowd-sensing network. Therefore, most previous works focus on the optimal participants selection strategy such that the selected participants can complete the required task assigned by an assessment center while the total rewards of selected participants are no larger than budget. Two works [47,48] propose a greedy algorithm to choose participants such that the selected participants can cover the points of interest while guaranteeing the sensing data can deliver successfully to the assessment center. Jia et al. [49] propose two budget feasible frameworks, Sensing Time Interval based Budget Feasible Framework(BFF-STI) and Bidding Time Interval based Budget Feasible Framework(BFF-BTI), to solve the problem of how to choose suitable smartphone users to maximize continuous time interval coverage under budget constraint. Zhang et al. in work [50], first design two functions to evaluate the works that can undertake query and sensing tasks simultaneously, and then introduce a novel spatial task assignment framework, called Spatial Recruiter, to maximize the coverage area of selected workers in hybridizing spatial crowdsourcing and crowd-sensing networks.

In addition to coverage quality, energy saving is another important concerning issue in the mobile crowd-sensing network. Lots of researchers save energy by three ways while guaranteeing the coverage quality. First, covering the region by adopting the low power sensors. For example, Cohn et al. [51] investigate how to sense human body motion by adopting an ultra-low power sensors. The second method is to reduce the energy consumption in the data delivery process. For instance, Philipp et al. [52] present a system to infer reading or sensing data for the positions even though there is no sensor deployed on it and in turn reduce the data transfer. Third, for the problem that the data connection may be intermittent when the network adopts a duty cycle for energy saving, Musolesi et al. [53] present an uploading strategy based on prediction mechanism which can calculate the current state when latest information is not available. Besides, Wang et al. [54] propose a data uploading framework to minimize the energy consumption and data cost when upload the sensing data. The framework named effSense can help different users to decide whether to offload or keep data in different scenarios.

As far as we known, the closest to our paper is the work done by Zhang et al. in [55]. Authors in [55] also investigate the problem of how to select the appropriate participants to cover some Points of Interest (POI) to sense some phenomenon of common interest such as providing place-related information and naming downtown places. However, there are two differences between our work and theirs [55]. The first one is the application scenario; our work is more suitable for monitoring the phenomenon of a whole critical region such as air quality monitoring or noise monitoring, since we take the sampling points whose locations are of uniform distribution inside the region, while the points of interest in the work [55] are randomly distribution. Furthermore, the sampling points in our work have the same contribution to the final monitoring results while the contribution of points of interest in [55] is different. The second difference is that our work introduces the concept of *coverage per reward* of each participant to evaluate the contribution of each participant to the whole coverage quality. In our greedy algorithm, we always choose the participant with maximum *coverage per reward* to improve the coverage quality and in turn minimize total rewards.

## 3. Discussion

### 3.1. Network Model

The critical region needed to provide air quality monitoring could be a tunnel or city. Without loss of generality, we assume that the region is a rectangle and the length and width of the region is *L* and *H*, respectively. In order to monitor the region environment, the sensors should collect the data at some positions located inside the region. Obviously, the number of such points is large and it is dificult to sense the physical information from each point in the region. Therefore, we choose some points in the region as *sampling points*. Assume that there are *m* sampling points in the region, we determine the locations of those *m* sampling points as following: the region is partitioned into *m* equal smaller rectangle regions where the length and width of the smaller region is $\frac{L}{m}$ and $\frac{H}{m}$, respectively. The centroid of each region is selected as a sampling point to represent the smaller region. Let $P=\{p_{1},p_{2},\ldots ,p_{m}\}$ denote the set of sampling points and the location of the *i*th sampling point is $p_{i}\left(x_{p_{i}},y_{p_{i}}\right)$. Furthermore, the air quality of each sampling point has the same contribution to the final air quality assessment results and the weighted of each point in the final result calculation is $\frac{1}{m}$.

**Figure 1:**
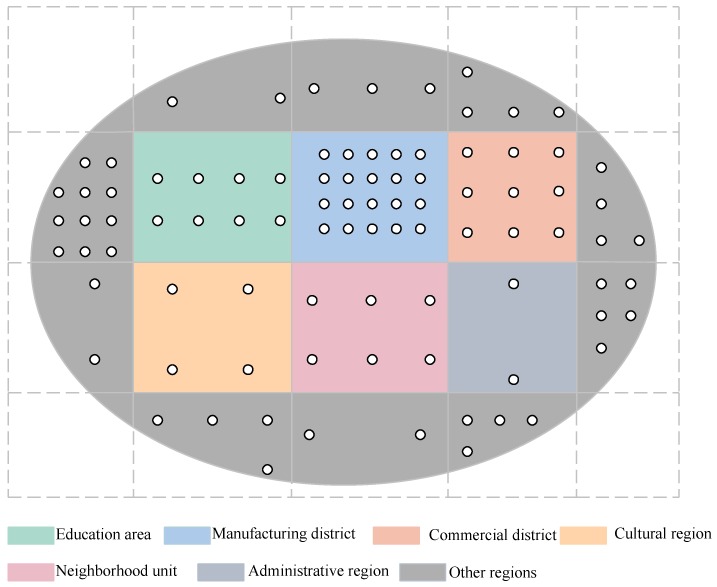
Illustration of a region divided into different sampling area.

Three points about the network model should be specified here: (1) the critical region could be of any type; it can be triangle, circle or even an irregular region. In other words, the size or the shape of the region are not limited. However, the large critical region can be partitioned into smaller region with regular region. (2) due to variability in sourced and geofeatures inside urban areas, the size of the small region is variable which is dependent on the inside sources and geographical characteristics. Corresponding, the contribution of the data collected from different smaller region may have different weight in making the final decision. (3) the sampling granularity in each smaller region is different which is also determined by the inside sources and geographical characteristics of the smaller region. For instance, if the critical region is an open plain without obvious pollution source, we will choose less sampling points to reduce the cost and data redundancy since the sampling data in such place won’t change dramatic. We use the equally partition strategy in each smaller region and the weight of each point in each smaller region is regarded as the same. Let us take an example shown in the Figure 1 to illustrate the partition process. We partition the whole region into 16 smaller regions. The air quality in a manufacturing district may be poorer than that of the education area since it has an air pollution source. Therefore, we take more sampling points in the manufacturing district and the weight of those collected data to the final quality assessment result is bigger compared to that from education area.

Assume that there are *n* participants carrying smart devices inside the region and all smart devices carried by the participants have the same physical character and the coverage region is a disk region with radius equal to $r_{s}$. Let *V* = {$v_{1},v_{2},\ldots ,v_{n}$} denote those participants, where the element $v_{j}\left(x_{v_{j}},y_{v_{j}}\right)$ denotes the instantaneous location of *j*th participant. The *j*th participant can collect the data from the *i*th sampling point if the distance between sampling point and participant is no larger than $r_{s}$. In other words, the *j*th participant can cover the *i*th sampling point when $d\left(v_{j},p_{i}\right)\leq r_{s}$. Thus:

(1) $$f_{v_{j}}\left(p_{i}\right)=\left\{\begin{array}{cc} 1 & d(v_{j},p_{i})\leq r_{s}\\ 0 & \mathrm{otherwise} \end{array}\right.$$

Due to the mobility of the participants, the location of each participant is flexible, the traverse paths of participants may be a regular curve or irregular curve. Assume that the initial location and terminal location of $v_{j}$ in the region are $I_{j}\left(x_{v_{j}}^{i},y_{v_{j}}^{i}\right)$ and $T_{j}\left(x_{v_{j}}^{t},y_{v_{j}}^{t}\right)$, respectively. Then the traverse path of $v_{j}$ is curve $\tilde{I_{j}T_{j}}$. The coverage region of the mobile participant $v_{j}$ is a belt region with the breadth of $2r_{s}$ and the center line is the traverse path $\tilde{I_{j}T_{j}}$. Obviously, $v_{j}$ can cover the sampling point if it is inside the coverage region of the *j*th participant. Let $C_{j}$ denote the set of sampling points covered by the participant $v_{j}$. We have:

(2) $$C_{j}=\left\{p_{i}|f_{v_{j}}\left(p_{i}\right)=1\mathrm{and}\;1\leq i\leq m\;\mathrm{and}\;v_{j}\in \tilde{I_{j}T_{j}}\;\right\}$$

**Figure 2:**
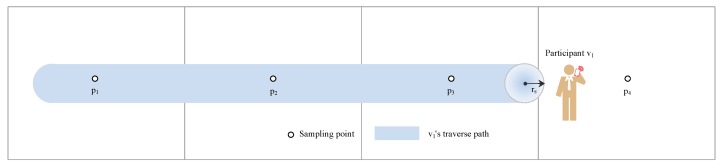
Illustration of the traverse path and the coverage region of the participant $v_{1}$.

The Figure 2 illustrates the traverse path of participant $v_{1}$ which covers three sampling points during the moving process. Thus, $C_{1}=\left\{p_{1},p_{2},p_{3}\right\}$.

With the popularity of smart devices, there are more than one active participants inside the critical region and one sampling point may be covered by different participants. The total number of covered different sampling points measures the coverage quality provided by a mobile crowd-sensing network. The goal of our work is to maximize the coverage quality in the critical region. Thus, covering maximum sampling points.

Note here, a participant completes the air quality task like this way: the participant opens the device embedded with sensing device to collect sampling data once it closes to the sampling point and uploads data through the 4G network or WIFI.

**Figure 3:**
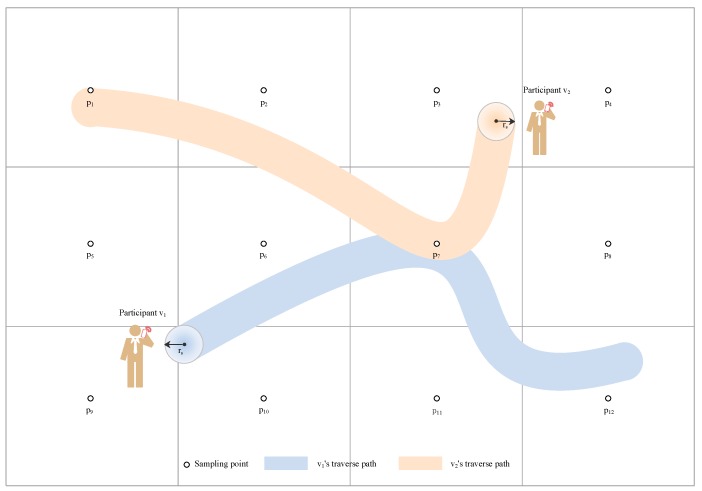
Illustration of a network example with two activity participants and 12 sampling points.

The Figure 3 shows a network example where there are 12 sampling points and two active participants in the critical region. During the process of movement, participant $v_{1}$ only covers one sampling point while $v_{2}$ covers two sampling points.

### 3.2. Communication Model

**Figure 4:**
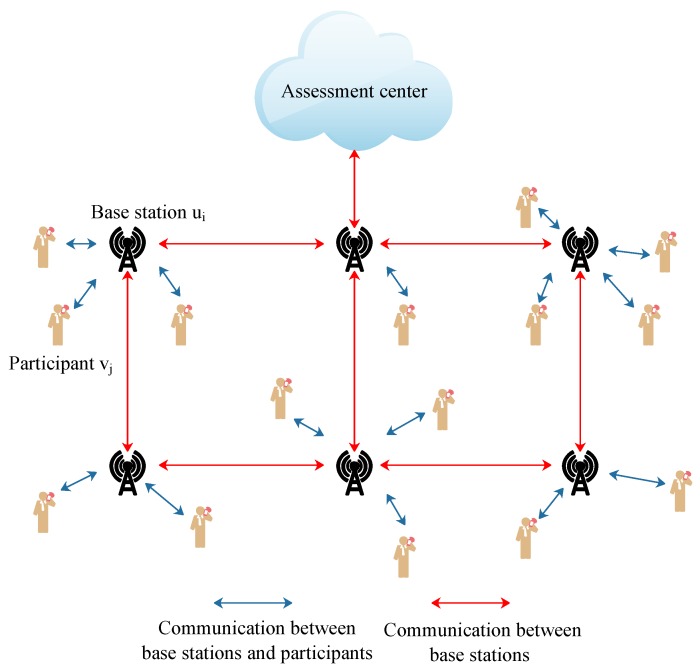
Illustration of connection between participant nodes and base stations.

The collected data should be transmitted to the assessment center. However, the assessment center location may be outside the communication range of the participants. Inspired by [56,57], we divide the participant nodes of a mobile crowd-sensing network into two types in order to transmit data more efficiently and reduce energy consumption: base stations and active participants carried with smart devices. At the begining, the assessment center will determine some base stations $u_{i}$ in the critical region which are used to collect data from the participants and retransmit data packets to the assessment center. When participants approach a base station, and satisfy the condition that $d\left(u_{i},v_{j}\right)\leq r_{c}$ ( $r_{c}$ is the radius of the communication range of *j*th participant), they will upload the collected data to a base station via Bluetooth or WIFI. Then, the base station will package all sensing data and forward the data packets to the assessment center by one-hop or multiple-hop. An communication model of the crowd-sensing network example is shown in Figure 4. This communication model can reduce the energy consumption that caused data transmitting and balance the workload for the cellular network.

### 3.3. Problem Description

As mentioned above, the goal of our work is to cover maximum sampling points in the critical region. The best way to solve this problem is to select maximum participants. However, it takes extra time and cost for participants when they complete the data collection task. They will ask some rewards which are decided according to the difficulty degree of completing the sampling task. Meanwhile, the total budget of the assessment center is also limited. Therefore, our work investigates the problem of how to maximize the coverage quality while the total rewards of selected participants is not larger than a predefined budget. We denote this problem as *Maximizing Coverage with Limited Budget (MCLB)* problem.

Let $B_{V}=\left\{b_{1},b_{2},\ldots ,b_{n}\right\}$ denote the set of rewards demanded by all participants. The reward follows a random distribution within the range $\left[B_{min},B_{max}\right]$. The maximum budget of the assessment center is *B*. Assume that the selected participants set is S(S⊂V). Then, the covered sampling points set is ${⋃}_{v_{j}\in S}C_{j}$. Suppose there are q(0<q≤m) different sampling points covered by set *S*. We use *W* measures the coverage quality provided by the crowd-sensing network and $W≐\frac{q}{m}$. Obviously, W=1 means that all sampling points are covered. Let indicator function $f(v_{j})$ indicate whether $v_{j}$ is selected or not, thus $f(v_{j})=1$ if $v_{j}$ is selected otherwise $f(v_{j})=0$. Then, the *MCLB* problem can be formulated as:

(3) $$\begin{array}{c} \mathrm{maximize}\;W=\frac{q}{m}.\\ \;s.t.\;\sum_{v_{j}\in S}f\left(v_{j}\right)b_{j}\leq B \end{array}$$

Obviously, the decision version of the *MCLB* problem is an NP hard problem: there exists a set of participants $S=\{v_{1},v_{2},\ldots ,v_{s}\}$ with the set of their covering sampling points *C* and corresponding required rewards $B_{v}=\left\{b_{1},b_{2},\ldots ,b_{s}\right\}$. Consider the predefined maximum budget *B* and $q_{o}$ sampling points needed to cover, we can verify the following problem in polynomial time whether (1) the total rewards of selected participants is larger than *B*; and (2) all $q_{o}$ sampling points have been covered by set *S*.

Accurately, we prove that the *MCLB* problem is an NP-Complete problem by transforming it into *knapsack problem* which has been proven as an NP-Complete problem [58]. The knapsack problem is described as follows. Given a set of items, each item has a different weight and value, we should determine the number of items which are added to a collection (knapsack) such that the total weight is less than a given limit and the total value is maximized. Compared to our work, the items are candidate participants, the value and the weight, respectively, correspond to sampling points covered by participants and the reward required by the participants. Therefore, the *MCLB* problem is an NP-Complete problem. We propose a greedy algorithm to solve the *MCLB* problem in the Section 4.

Table 1 notates the symbols used in our paper.

## 4. Materials and Methods

In this section, we propose a greedy algorithm to solve the *MCLB* problem.

The main idea of greedy algorithm can be summarized as: we introduce the *coverage per reward* $e_{j}$ as selection criterion of participants, where coverage per reward of the participant $v_{j}$ is defined as the cost of assessment center should pay for participant to cover one sampling point. Thus, $e_{j}=\frac{y}{b_{j}}$, where *y* is the size of set $C_{j}$. The larger $e_{j}$ is, the more efficiency the participant $v_{j}$ will has.

The greedy algorithm includes three steps:

Firstly, for the participants set *V*, we eliminate the participant whose reward is larger than the maximum budget *B*. Let $V^{\prime }=\left\{v_{1},v_{2},\ldots ,v_{n^{\prime }}\right\}$ denote the remaining participants set and the number of sampling points covered by $v_{j}$ is $y_{j}$. Then we calculate the coverage per reward of each participant in the set $V^{\prime }$, we have, $E=\{\frac{y_{1}}{b_{1}},\frac{y_{2}}{b_{2}},\prime ,\frac{y_{n^{\prime }}}{b_{n^{\prime }}}\}$. We select the participant $v_{max}$ with the maximum coverage per reward $e_{max}$ into the set *S* and remove the participant $v_{max}$ from $V^{\prime }$. Thus, $V^{\prime }=V^{\prime }\backslash v_{max}$ and the selected participants set $S=S\cup v_{max}$.

**Figure 5:**
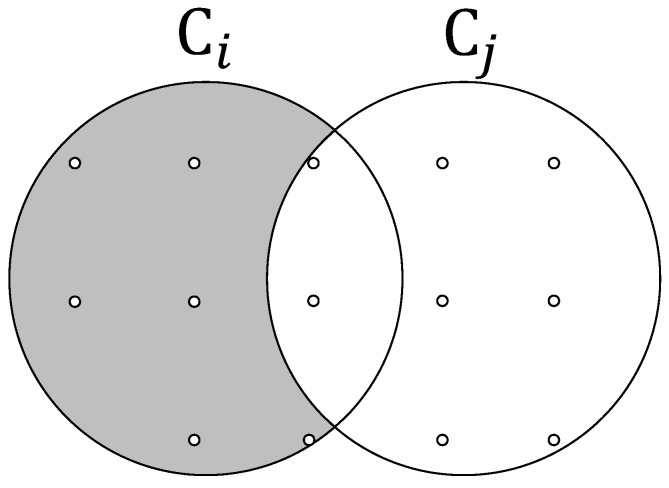
Illustration of how to compute the coverage per reward if two participants cover the some same sampling points.

Secondly, calculate the coverage per reward of the elements in the set $V^{\prime }$ again. The calculation has little difference from that in the first step since the elements in $V^{\prime }$ may cover the same sampling points with the selected participants in set *S*. For each element $v_{i}$ in the set $V^{\prime }$, we first determine the number of sampling points which are covered by $v_{i}$ while not covered by the participants in set *S*. Then the coverage per reward of $v_{i}$ can be computed by $\frac{y_{i}-y_{common}}{b_{i}}$. We take an example to explain how this process works. The Figure 5 shows the coverage regions of two participants $v_{i}$ and $v_{j}$, where $v_{i}\in V^{\prime }$ and $v_{j}\in S$. Those two participants cover two same sampling points, then the coverage per reward of $v_{i}$ is $\frac{8-2}{0.1}$. Again, we select the participant with maximum cost efficiency $v_{max}$ in set $V^{\prime }$ into *S* and remove that participant from set $V^{\prime }$. If $R+b_{max}>B$, we choose participant with the second largest coverage per reward, and it terminates if there are no participants satisfied condition.

Thirdly, goes to the second step until one of following conditions is satisfied: (1) all sampling points are covered, thus W=1; (2) the total rewards of selected participants set *S* is equal to *B*; (3) the remain set $V^{\prime }$ is empty; (4) the reward of each participant in set $V^{\prime }$ add *R* is larger than *B*. The detail of the greedy algorithm is shown in Algorithm 1.

**Algorithm 1** Greedy algorithm for MCLB problem**Require:** Sampling points set *P*; participants set *V* and corresponding rewards set $B_{V}$; the selected participants set S=∅; total rewards of selected participants R=0; limited budget *B*; **Ensure:** The set of selected participants *S*, the coverage quality *W* of this case;**1:** **For all $v_{i}\epsilon V$ do;****2:** **if $b_{i}>B$;****3:** **V⟵V\$v_{i}$;****4:** **For all $v_{i}\epsilon V$ do;****5:** **$e_{i}$=$y_{i}/b_{i}$;****6:** **select $v_{max}\epsilon V$ with maximum $e_{max}$;****7:** **$S=S\cup \{v_{max}\}$;****8:** **$R=R+b_{max}$;****9:** **repeat****10:** **    $V⟵V\backslash v_{max}$;****11:** **    if $b_{i}\leq \left(B-R\right)$****12:** **    select $v_{i}\epsilon V$ with maximum $\frac{y_{i}-y_{common}}{b_{i}}$;****13:** **    $S=S\cup \{v_{i}\}$;****14:** **    $R=R+b_{i}$;****15:** **until W=1 or R≥B or V=∅;**

Complexity analysis: Assume that there are in total *n* active participants in the region. Obviously, most complicated case is that all active participants are selected and the complexity is $O(n^{2})$. Which means that the complexity of the greedy algorithm increase with the number of active participants in the region. In order to reduce the complexity, we adopt partition strategy when the critical region is larger. Thus, we divide big critical region such as city into different size smaller regions according to sources inside or geographical characteristics. For each smaller region, the assessment center recruit participants active in the smaller region and select the most appropriate participants set to collect the data. All collected data are sent to a base station in each smaller regions which is assigned by assessment center. The base station transmits the collected data to the assessment center by one-hop or multi-hop. As we can see the complexity will drop significantly based on such partition process since the complexity in such case is determined by the smaller region with the maximum number of candidate participants. Thus, $O(n_{max}^{2})$, where $n_{max}<n$.

## 5. Results

In this section, we compare the performance of our proposed algorithm with the one computed by the algorithm proposed by Zhang et al. [55], the main idea of the algorithm proposed by Zhang first enumerates all subsets of participants with *k* elements and then complements these subsets using the modified greedy algorithm.

### 5.1. Simulation Setting

Assume that the critical region is a square area with size of 3000 m × 3000 m. The sampling points are determined by the following steps: the protected region is divided into multiple lattices and select the center point of each lattice as the sampling point. In real application, the traverse paths of the selected participants can be any kinds of curves. In our simulation, we simulate the traverse paths of all mobile participants based on random walking: first, assume that the initial location of each participant follows a uniform distribution in the region; next, the participants change direction after walking every 100 s, we also assume that the walking speed of each participant is the same and equal to 1.5 m/s. If the distance between the sampling point and the traverse path of one participant is smaller than $r_{s}$, the participant can collect the data from the sampling points. In other words, the participant can cover the sampling point. The reward of each participant follows a random distribution within the region $\left[B_{min},B_{max}\right]$, where $B_{min}=0$ and $B_{max}=0.1$.

**Figure 6:**
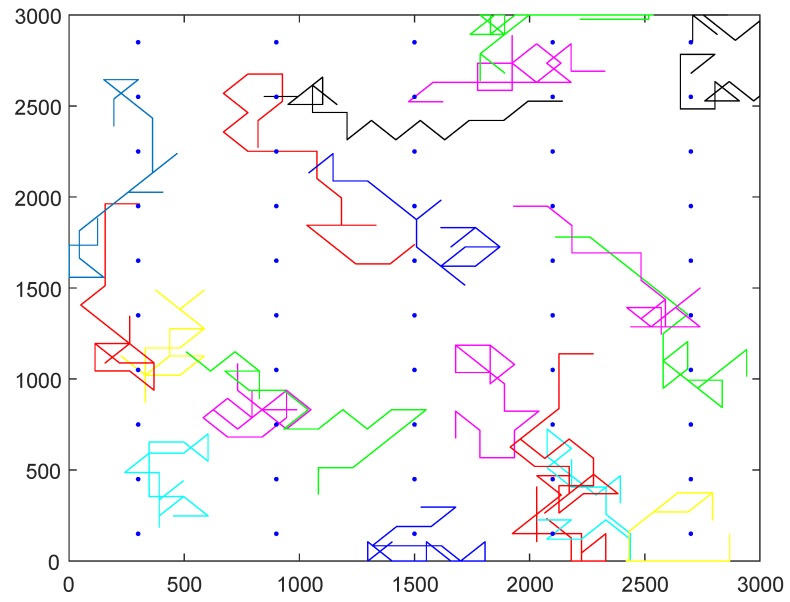
Illustration of a simulation network example.

Figure 6 illustrates a simulation network example; there are 50 sampling points denoted by blue points and 20 participants whose traverse paths are denoted by different continuous curves in the region. In the simulation experiments, we compare the performance of our greedy algorithm with Zhang’s algorithm.

### 5.2. Simulation Results

**Figure 7:**
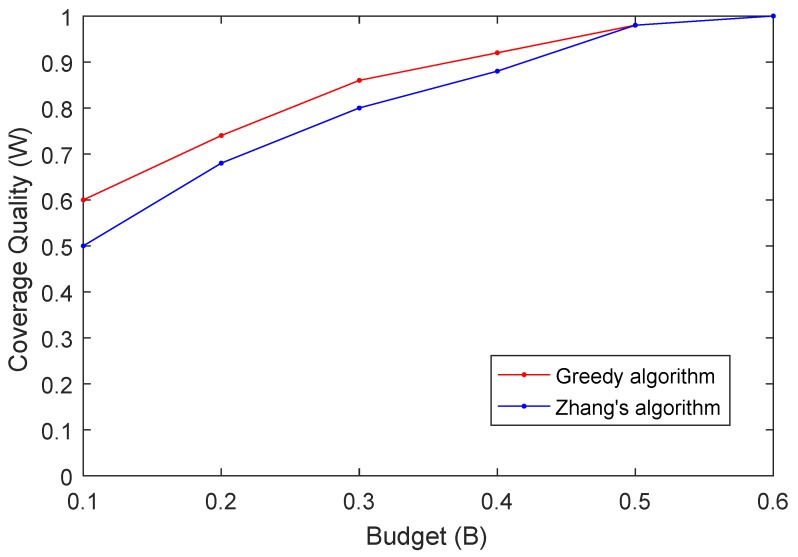
Coverage Quality (W) vs. Budget (B).

In the first experiment, we investigate the relation between the coverage quality *W* and the limited budget *B*. In this experiment, we set the radius of participant as $r_{s}=30$, the number of sampling points m=50, and the number of participants n=20. The budget *B* varies from 0.1 to 0.6 with 0.1 as step size. The simulation results are shown in Figure 7. As we can see from the results, the coverage quality *W* increases with the increases of the budget *B*. This is because the more the budget is, more participants can be selected to cover the sampling points. We should point out that when the budget is larger than some threshold value, all active participants may be selected to cover the sampling points since the assessment centers have enough budget. In such a case, the result computed by our proposed algorithm is the same as the one computed by Zhang’s algorithm as shown in the Figure 7. The data indicate that our greedy algorithm outperforms Zhang’s algorithm when considering budget constraints, since our greedy algorithm always chooses the participant who can cover more sampling points with less reward in each step.

**Figure 8:**
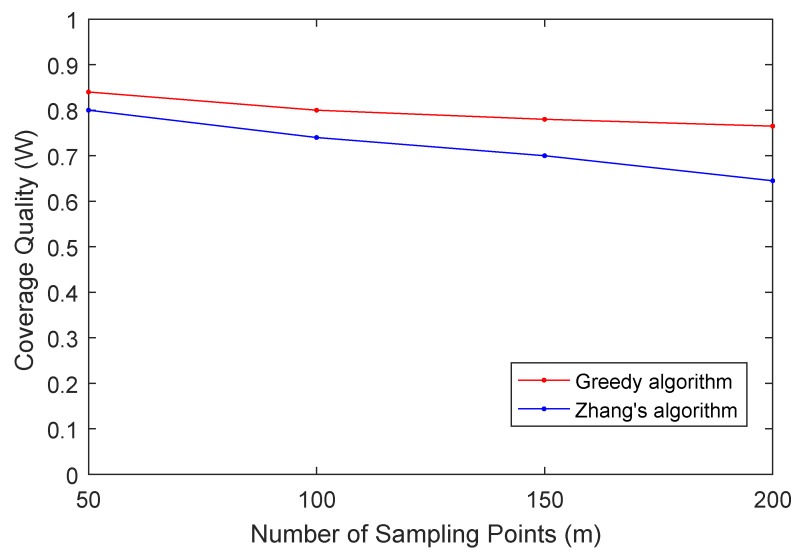
Coverage Quality (W) vs. Number of Sampling Points (m).

In the second experiment, we study how the number of sampling points *m* affects the coverage quality *W*. In this experiment, we set B=0.3, $r_{s}=30$, n=20 and vary *m* from 50 to 200 with 50 as step size and the data shown in the Figure 8. As we can see from the figure, it is not unexpected that the coverage quality *W* decreases with the increases of the number of sampling points *m*. The reason is that the bigger parameter *m* means the higher precision of the environmental quality monitoring that is required. More sampling points need to be covered while the candidate participants and budget do not increase. Therefore, the coverage quality decreases.

**Figure 9:**
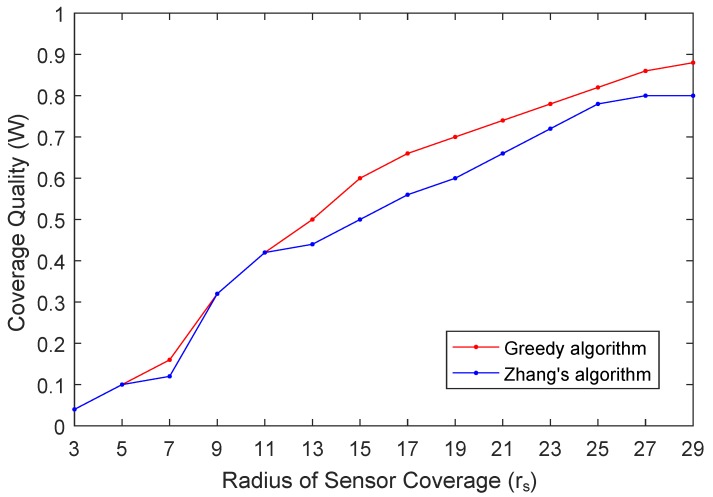
Coverage Quality (W) vs. Radius of Sensor Coverage $\left(r_{s}\right)$.

In the third experiment, we investigate how the sensor coverage radius $r_{s}$ impacts the coverage quality *W*. In this experiment, we set B=0.3, m=50 and n=20. The results are shown in Figure 9. As the results shown, the coverage quality *W* has a growth trend as the coverage radius $r_{s}$ increases. The reason for this result is that with the same spacing between adjacent sampling points, the larger the sensor coverage radius $r_{s}$ is, the larger the coverage region of participant is, and in turn more sampling points can be covered. It is worth noting that our proposed algorithm and Zhang’s algorithm achieve almost the same results when the coverage radius of participants is small. In such cases, the algorithm proposed by Zhang compute the result from enumeration method and complement with modified greedy algorithm, while our proposed algorithm selects participants according to their coverage per reward. When the coverage radius becomes larger, the results computed by the algorithm proposed by Zhang are achieved by the enumeration method which chooses *k* subsets randomly while our proposed methods choose the one with maximum cost efficiency, and then the results are worse than that of our proposed algorithm.

**Figure 10:**
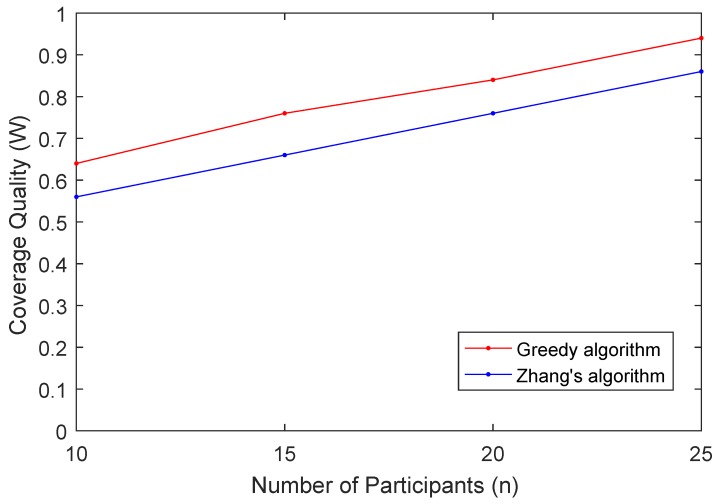
Coverage Quality (W) vs. Number of Participants (n).

In the last experiment, we examine how parameter *n* affects the coverage quality *W*. In this experiment, we set B=0.3, $r_{s}=30$, m=50 and vary *n* from 10 to 25 with 5 as the step size. The results are shown in Figure 10. The data indicate that the coverage quality *W* increases with the increases of the number of participants *n*. Bigger *n* means there are more active participants in the region. There are more candidate participants for the greedy algorithm to select. In other words, the algorithm can find the participants with smaller required reward and in turn achieve maximum coverage quality with less money. In general, our proposed algorithm outperforms the algorithm proposed by Zhang.

It can be seen from the above simulation results and analysis that the budget *B*, the number of sampling points *m*, the coverage radius of sensor $r_{s}$ and the number of participants *n* have significant influence on the coverage quality *W*. We should set each parameter according to the monitoring requirement of the critical region when our proposed algorithm is applied in a different scenario.

## 6. Conclusions

In this paper, we have studied the problem of how to maximize the coverage quality with limited budget in a mobile crowd-sensing network when it applies to monitor the critical region environment. We first divide critical regions into multiple smaller rectangle regions, and all centroids of the rectangle regions are selected as sampling points. The quality of sampling points represents the quality of the whole region. In order to cover maximum sampling points, we have proposed a greedy algorithm to select suitable participants to cover maximum sampling points while the total rewards paid for selected participants is no larger than the limited budget. The simulation results have validated the effectiveness of the proposed algorithm. Our work gives an important insight into practical participants selection of mobile crowd-sensing networks for monitoring the phenomenon of the whole critical region.

## Figures and Tables

**Table 1:** Summary of main symbols.

Symbol	Description
*m*	number of sampling points
*H*	width of sampling area
*L*	length of sampling area
$p_{i}$	the *i*th sampling point
*P*	set of sampling points
*n*	number of participants
$u_{l}$	the *l*th base station
$r_{c}$	radius of communication range of participant
$v_{j}$	the *j*th participant
*V*	set of participants
$r_{s}$	sensing radius of pariticipants
$I_{j}$	initial location of $v_{j}$
$T_{j}$	terminal location of $v_{j}$
$\tilde{I_{j}T_{j}}$	traverse path of $v_{j}$
$C_{j}$	set of sampling points covered by the traverse path of $v_{j}$
$b_{j}$	reward of $v_{j}$
$B_{V}$	set of the rewards demanded by all participants
*B*	maximum budget
*S*	set of selected participants
*W*	coverage quality provided by selected participants set *S*
